# Genotyping-by-sequencing reveals three QTL for clubroot resistance to six pathotypes of *Plasmodiophora brassicae* in *Brassica rapa*

**DOI:** 10.1038/s41598-017-04903-2

**Published:** 2017-07-03

**Authors:** Fengqun Yu, Xingguo Zhang, Gary Peng, Kevin C. Falk, Stephen E. Strelkov, Bruce D. Gossen

**Affiliations:** 1 Agriculture and Agri-Food Canada, Saskatoon Research and Development Centre, 107 Science Place, Saskatoon, SK S7N OX2 Canada; 2 Department of Agricultural, Food and Nutritional Science, University of Alberta, Edmonton, AB T6G 2P5 Canada

## Abstract

Clubroot, caused by *Plasmodiophora brassicae*, is an important disease of Brassica crops worldwide. F_1_ progeny from the *Brassica rapa* lines T19 (resistant) × ACDC (susceptible) were backcrossed with ACDC, then self-pollinated to produce BC_1_S_1_ lines, From genotyping-by-sequencing (GBS) of the parental lines and BC_1_ plants, about 1.32 M sequences from T19 were aligned into the reference genome of *B*. *rapa* with 0.4-fold coverage, and 1.77 M sequences with 0.5-fold coverage in ACDC. The number of aligned short reads per plant in the BC_1_ ranged from 0.07 to 1.41 M sequences with 0.1-fold coverage. A total of 1584 high quality SNP loci were obtained, distributed on 10 chromosomes. A single co-localized QTL, designated as *Rcr4* on chromosome A03, conferred resistance to pathotypes 2, 3, 5, 6 and 8. The peak was at SNP locus A03_23710236, where LOD values were 30.3 to 38.8, with phenotypic variation explained (PVE) of 85–95%. Two QTLs for resistance to a novel *P*. *brassicae* pathotype 5x, designated *Rcr8* on chromosome A02 and *Rcr9* on A08, were detected with 15.0 LOD and 15.8 LOD, and PVE of 36% and 39%, respectively. Bulked segregant analysis was performed to examine TIR-NBS-LRR proteins in the regions harboring the QTL.

## Introduction

The soil-borne pathogen *Plasmodiophora brassicae* Woronin causes clubroot disease in Brassica oilseed and vegetable crops worldwide. It belongs to the Infra Kingdom Rhizaria, which is a diverse group of amoeboid protists^1^. The haploid resting spores of *P*. *brassicae* release zoospores that infect root hairs, in which multi-nucleate plasmodia are formed. These plasmodia develop into uninucleate secondary zoospores, which are released into the soil and then infect young roots. Secondary multi-nucleate plasmodia develop rapidly and colonize the root cortex^2^, which stimulates the host plants to produce the characteristic root galls, known as clubs. Disorganization of tissues in the clubbed roots restricts the flow of water and nutrients, resulting in above-ground symptoms that include stunting, yellowing, premature senescence, and reduction in both seed yield and quality^3^. The plasmodia become sessile and divide to produce resting spores, which are released from decaying clubs into the soil, where they can survive for many years^2^. The combination of prolonged survival in the absence of a wide host range including many weed species, and relative insensitivity to most reduced-risk fungicides and bactericides makes it difficult to manage clubroot using crop rotation or anti-microbial seed treatments^4^. Therefore, genetic resistance is generally considered to be the most effective approach for clubroot management.

Clubroot is a serious constraint to canola (*Brassica napus* L.) production on the Canadian prairies. In Canada, five pathotypes of *P*. *brassicae* (pathotypes 2, 3, 5, 6 and 8) have been identified based on the differential system of Williams^5^, with pathotype 3 the most prevalent on canola in the prairie region^6–8^. The first clubroot-resistant canola cultivar in western Canada was released in 2009, and was followed by the release of other resistant cultivars from various seed companies starting in 2010. These cultivars exhibited strong resistance to the pathotypes of *P*. *brassicae* present in Canada^9^. However, resistance in Canadian canola cultivars has been overcome recently by new strains of the pathogen identified in Alberta^10^. These new strains have been informally designated as pathotype 5x because they are classified as pathotype 5 on the differentials of Williams (1966)^5^ but (unlike the original pathotype 5) are highly virulent on clubroot resistant canola.

Lines with resistance to a broad range of pathotypes of *P*. *brassicae* have been identified in the canola progenitor species *B*. *rapa* ^11, 12^. This species could be used to broaden the genetic base of clubroot resistance in canola. Introgression of traits from *B*. *rapa* into canola through interspecific crosses is possible^13–17^, so resistance to clubroot from *B*. *rapa* could be transferred into canola through conventional breeding.

The identification and genetic mapping of clubroot resistance genes has been carried out in *B*. *rapa* ^18–34^, *B*.*oleracea* ^35–44^ and *B*. *napus* ^45–51^. Two resistance genes, *CRa* and *Crr1*, have been isolated from Chinese cabbage lines of *B*. *rapa*. They encode Toll-Interleukin-1 receptor/nucleotide-binding site/leucine-rich-repeat (TIR-NBS-LRR, TNL) proteins^52, 53^.

Genotyping-by-sequencing (GBS) offers a new tool to explore the genetic control of complex traits. GBS analysis was used in the current study to: 1) characterize genome-wide variants in *B*. *rapa*; 2) identify SNP sites that could be used for genetic mapping; and 3) detect QTL effectively resistant to multiple pathotypes of *P*. *brassicae* identified in western Canada; and 4) identify possible candidate genes for each QTL.

## Results

### Resistance to clubroot in *B*. *rapa* canola line T19 and BC_1_S_1_ progenies

**Figure 1:**
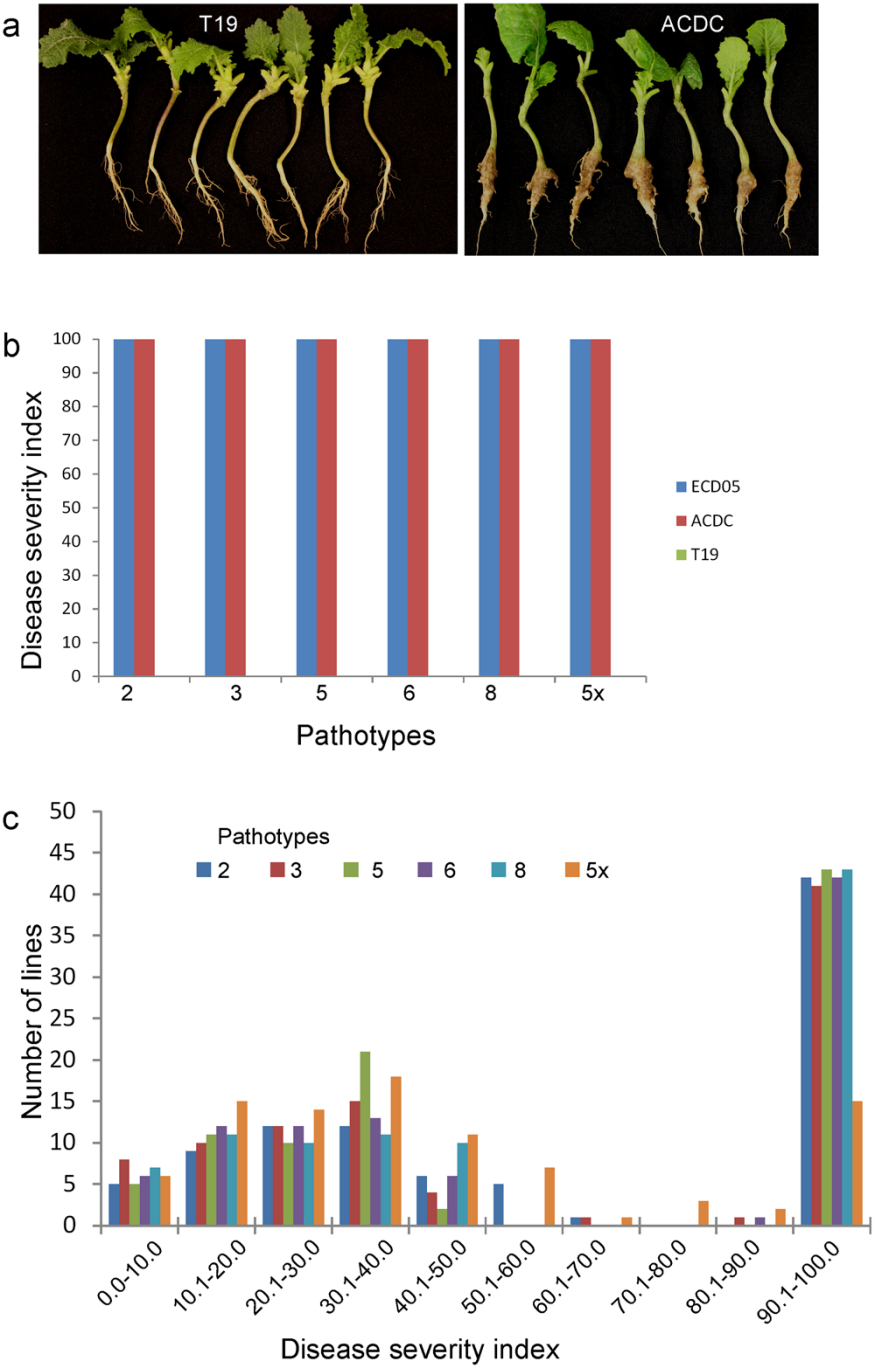
Evaluation of parental lines and BC_1_S_1_ population derived from ACDC × (ACDC × T19) for resistance to clubroot. (**a**) R and S phenotypes in the parental lines with pathotype 3; (**b**) Disease severity indexes (DSIs) of the parental line and susceptible control ECD 05 with pathotype 2, 3, 5, 6, 8 and P5x of *Plasmodiophora brassicae*; (**c**) Distribution of DSIs in the BC_1_S_1_ population consisting of 92 lines.

The *B*. *rapa* canola line T19 was shown to be highly resistant to pathotype 3 while ACDC was highly susceptible (Fig. 1a). To determine if T19 was resistant to the other pathotypes present in Canada, we inoculated T19 plants with six Canadian pathotypes of *P*. *brassicae*. This line was found to be highly resistant to all of the pathotypes (0% DSI), while ACDC and *B*. *rapa* subsp. *pekinensis* (European Clubroot Differential (ECD) 05, a susceptible check) were highly susceptible (100% DSI) (Fig. 1b).

Ninety two BC_1_S_1_ lines derived from 92 BC_1_ plants were then tested for resistance to each of the pathotypes. All seedlings of the susceptible check (ECD 05) and the susceptible parent ACDC developed severe clubbing (100% DSI). The resistant parent, T19, was resistant to each pathotype (0% DSI, Supplementary Fig. S1), confirming that T19 was highly resistant. The distribution of clubroot severity in response to inoculation with pathotypes 2, 3, 5, 6 and 8 (Fig. 1c, Supplementary Fig. S1) could be divided into two major classes: resistant (R) lines with DSI < 60% and susceptible (S) lines with DSI > 60%. Of the 92 lines, 49 lines were classified as R and 42 lines as S to each of the pathotypes (Fig. 1c). The segregation of R and S fitted a 1:1 ratio (X^2^ = 0.39; *P* = 0.53), indicating that resistance to the pathotypes may be controlled by a single resistance gene in the R parent line T19. DSIs in the population were highly correlated (r ≥ 0.92) among the five pathotypes (Table 1), indicating that resistance to the pathotypes may be controlled by the same gene or closely linked genes. However, 71 of the 92 lines were classified as R and 21 lines as S to pathotype 5x. Segregation for R and S in the population did not fit a 1:1 ratio (X^2^ = 27.2; *P* < 0.001), but fit a 3:1 ratio (X^2^ = 0.23; *P* = 0.63). This indicates that resistance to pathotype 5x may be controlled by two resistance genes in T19. A correlation of the DSIs in response to 5x with those to the rest of the pathotypes was not found (r ≤ 0.06) (Table 1), indicating that the genes for resistance to pathotype 5x are not linked to the gene for resistance to pathotypes 2, 3, 5, 6 and 8.

**Table 1:** Correlation coefficients among the disease severity index values after inoculation of BC_1_S_1_ families derived from ACDC × T19 for resistance to six pathotypes of *Plasmodiophora brassicae*.

Pathotype	3	5	6	8	5x
2	0.93**	0.94**	0.94**	0.92**	−0.03
3		0.95**	0.95**	0.95**	0.06
5			0.96**	0.96**	0.02
6				0.96**	0.03
8					0.04

**Significance level at P < 0.01.

### Alignment of DNA short reads into the *B*. *rapa* reference genome

Approximately 1.32 million (M) sequences were aligned into the chromosomes of the reference genome in the resistant parent T19 and 1.77 M sequences in the susceptible parent ACDC (Table 2). The accumulated length of sequences was 98.3 Mb with 0.4-fold coverage in T19, and 131.1 Mb with 0.5-fold coverage in ACDC. The average aligned short read length was 74.3 bases in T19 and 74.5 bases in ACDC (Table 2).

**Table 2:** Alignment of short reads and identification of variants in the parents and 92 BC_1_ individuals.

Materials		Number of sequences (x 10^3^)	Accumulated length of sequences (bases × 10^3^)	Fold coverage	Number of bases/sequence	Number of variants (x10^3^)	SNP (%)	InDel (%)
Parents	T19	1324	98.3	0.4	74.3	126.3	87.9	12.1
	ACDC	1769	131.8	0.5	74.5	162.1	87.9	12.1
BC_1_	Mean ± SE	438 ± 29	33.4 ± 2.2	0.1 ± 0	76.4 ± 0.1	55.3 ± 3.0	88.1	11.9
	Total	40280	3076.4	12.0		5094.4	88.1	11.9

**Figure 2:**
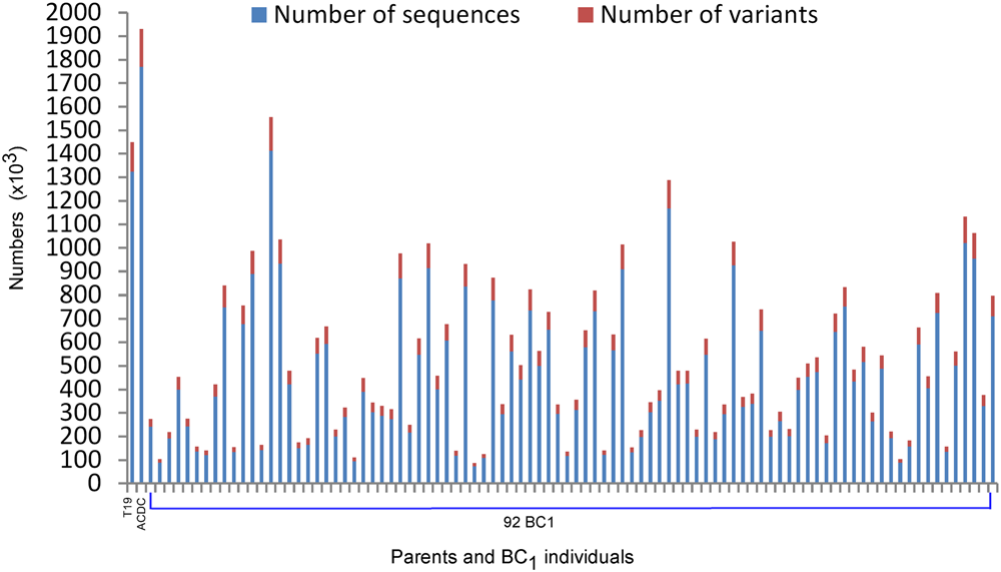
Numbers of sequences aligned into the *Brassica rapa* reference genome and variants (SNPs and InDels) identified in each sample in comparing with the DNA sequence of the reference genome.

The number of aligned short reads from each sample in the BC_1_ population varied, ranging from 0.07 to 1.41 M sequences (Fig. 2). The accumulated length of sequences in the 92 samples was 3076.4 Mb with 12.0-fold coverage. However, the average accumulated length of sequences was 33.4 Mb with 0.1-fold coverage (Table 2).

### Identification of variants in the population

There was a strong positive correlation (r = 0.99) between the numbers of variants compared with the number of sequences aligned into the reference genome (Fig. 2), indicating that the number of variants identified each sample was associated with sequencing depth. However, the proportions of SNPs and InDels were similar in the parental lines and BC_1_ population, with about 88% and 12%, respectively (Table 2, Supplementary Fig. S2). Variants in both T19 and ACDC samples were frequent, with 126.3 K and 162.1 K respectively (Table 2). The numbers of variants in the BC_1_ population were in a range of 14 to 143 K (Fig. 2) with a mean of 55.3 K variants per sample (Table 2).

### Identification of polymorphic SNP sites in the BC_1_ population and construction of linkage groups

**Figure 3:**
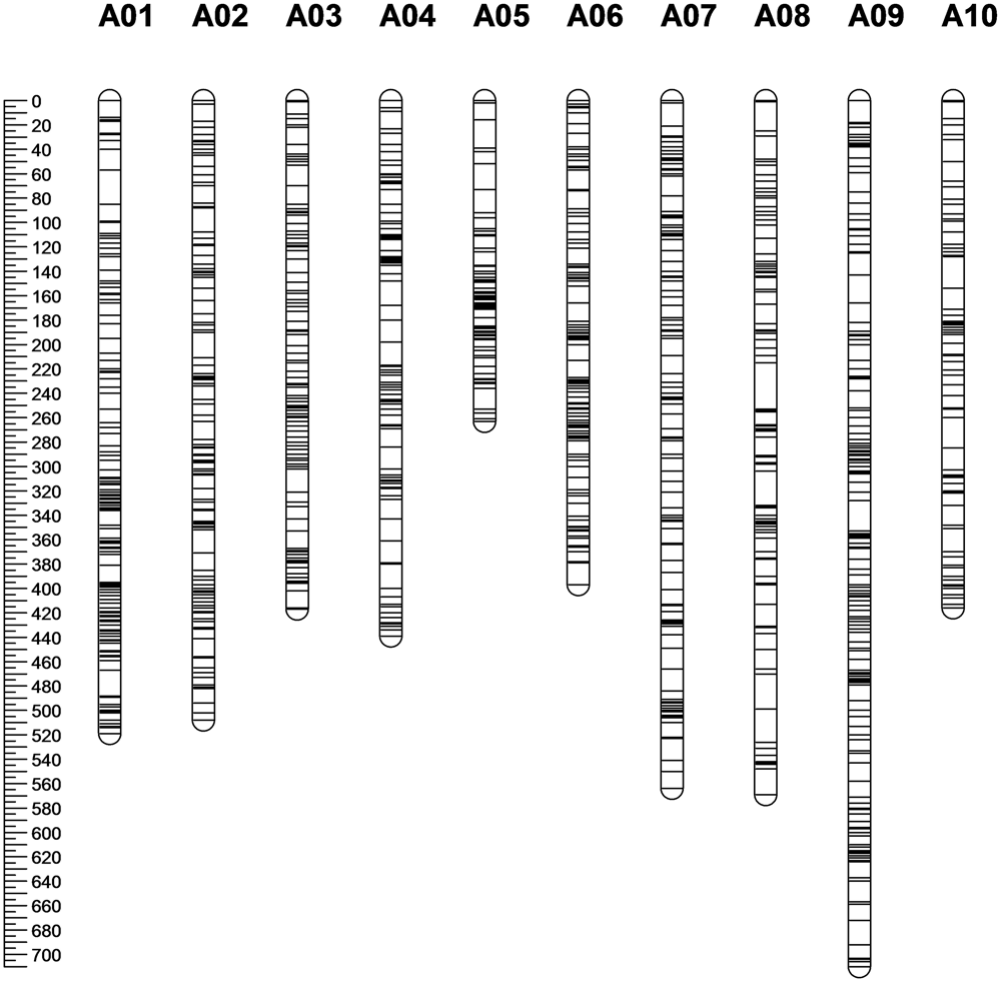
The linkage map of *B*. *rapa* consisting of 1584 SNP sites.

There were 16,618 SNPs and 2,127 InDels identified in at least 46 of the 92 BC_1_ samples (50% of the samples). Since the susceptible parent, ACDC, is a doubled haploid line, all SNP sites should theoretically be homozygous. However, out of 16,618 SNP sites, 3,263 SNP sites (19.6%) had a heterozygous genotype. Monomorphic phenotypes between the parents or among the 92 individuals were identified in 8,392 SNP sites (50.5%) (Supplementary Table S1). The remaining 4963 SNP sites were further analyzed using software JoinMap 4.1. A total of 1584 SNP sites, accounting for 9.5% of 16,618 SNP sites, could be assigned into 10 chromosomes of *B*. *rapa*. A genetic map consisting of 1584 SNP sites distributed to 10 chromosomes of *B*. *rapa* was constructed (Fig. 3). The number of SNP sites was in a range of 120 to 219, with an average number of 157 on each chromosome in the map. The length of each chromosome ranged from 263.15 to 709.51 cM with an average length of 479.3 cM. The SNP interval of each chromosome ranged from 2.07 to 3.98 cM with an average of 3.05 cM (Supplementary Table S2).

### Identification of QTL for resistance to six pathotypes of *P*. *brassicae*

**Figure 4:**
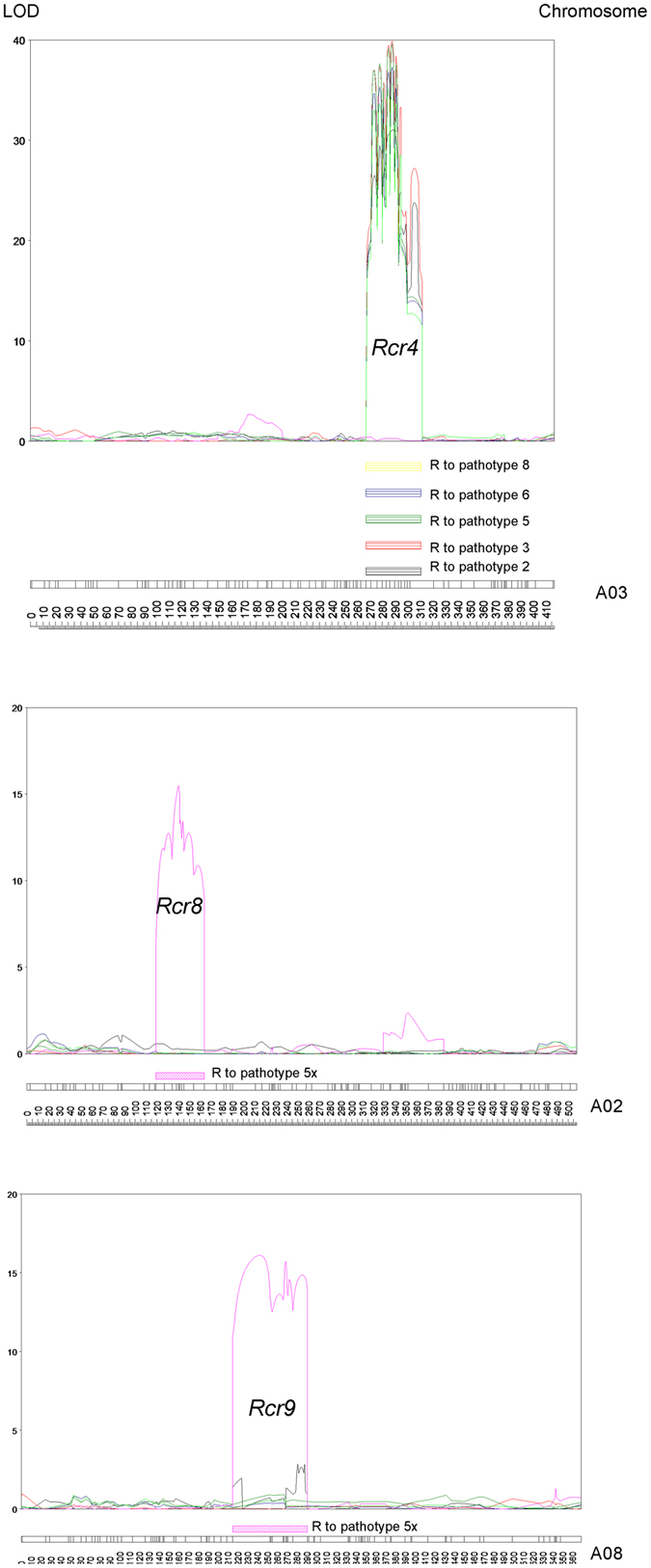
Three significant QTL of *Rcr4*, *Rcr7* and *Rcr8* detected chromosomes on A03, A02 and A08, respectively.

Mapping of the QTLs was performed using the linkage map (Fig. 3) and trait values for resistance to the six pathotypes of *P*. *brassicae*. QTL mapping analysis resulted in three QTLs being detected in chromosomes A02, A03 and A08 (Table 3). A single co-localized QTL, designed as *Rcr4*, was detected for resistance to pathotypes 2, 3, 5, 6 and 8, but not to pathotype 5x, which was located on chromosome A03 (Fig. 4). *Rcr4* spanned from 267.48 to 310.75 cM with a peak position at 282.65 cM. The SNP site nearest the peak for *Rcr4* was at SNP site A03_23710236 (chromosome_physical location) with LOD values of 30.3 to 38.8 for the five pathotypes, and phenotypic variation explained (PVE) 85% to 94% (Table 3). Two QTLs, designated *Rcr8* and *Rcr9*, were detected on chromosomes A02 and A08, respectively, for resistance to pathotype 5x. *Rcr8* spanned from 126.91 to 164.4 cM, with a peak position at 139.8 cM. The SNP site nearest the peak for *Rcr8* was at SNP site A02_18552018 (15 LOD), with PVE of 36%. *Rcr9* spanned from 214.61 to 291.25 cM, with a peak position at 242.5 cM. The SNP site nearest the peak for *Rcr9* was at A08_10272562 (15.8 LOD) with PVE of 39% (Table 3). As show in Table 3, the values of additive for the three QTL were positive, indicating that the resistant loci were derived from the resistant parent T19.

**Table 3:** QTL position and phenotypic variation explained (PVE) for resistance to six pathotypes of *Plasmodiophora brassicae* in *Brassica rapa* line T19.

QTL	Pathotype	Chr	Interval (cM)	Threshold LOD	Peak				Nearest SNP to peak	
					LOD	Position (cM)	PVE (%)	Additive	Name	Position
*Rcr4*	2	A03	267.48–310.75	4.09	30.3	289.7	85	69.4	A03_23710236	282.65
*Rcr4*	3	A03	267.48–310.75	4.15	38.8	288.1	92	75.1	A03_23710236	282.65
*Rcr4*	5	A03	267.48–310.75	4.61	38.5	288.3	94	72.7	A03_23710236	282.65
*Rcr4*	6	A03	267.48–310.75	4.03	36.4	288.3	93	73.8	A03_23710236	282.65
*Rcr4*	8	A03	267.48–310.75	4.03	34.6	288.1	93	73.2	A03_23710236	282.65
*Rcr8*	5x	A02	126.91–164.4	4.14	15.0	139.8	36	46.3	A02_18552018	139.93
*Rcr9*	5x	A08	214.61–291.25	4.14	15.8	242.5	39	38.5	A08_10272562	253.15

### Identification of TNL genes and variants in the genes in the target regions

DNA short reads from selected BC_1_ individuals, based on clubroot severity and the presence of SNP alleles in the respective identified QTL peaks, were pooled and aligned into the reference genome (Supplementary Table S3). The TNL genes in the target regions were identified (Table 4). The numbers of poly variants in the TNL genes of the *Rcr4*, *Rcr8* and *Rcr9* intervals were assessed because the poly variants represent differences in the DNA sequences between the R and S bulks. SeqMan Pro software was used to sort variants that affected amino acid sequences into four groups: non-synonymous, nonsense, frameshift and synonymous variants. However, nonsense and frameshift variants were not found in any of the TNL genes in this study.

**Table 4:** Number of variants identified in TNL genes in the target regions.

QTL/Chr	Gene	Location	No of variants^1^			Number of sequences	
			Sy	NS	Total	R bulk	S bulk
*Rcr4*/A03	Bra012541	23717282 … 23721752	4	0	4	237	139
	Bra019413	24350950 … 24353977	1	3	4	634	34
	Bra019412	24370531 … 24371199	0	0	0	8	2
	Bra019410	24373815 … 24379176	3	1	4	119	91
	Bra019409	24381590 … 24386315	1	0	1	64	59
	Bra019273	25345715 … 25352019	0	0	0	73	58
*Rcr8*/A02	Bra022069	18684900 … 18686777	0	0	0	0	0
	Bra022071	18690084 … 18692787	0	0	0	14	26
	Bra026556	20525858 … 20529292	0	0	0	1	7
	Bra032996	22083981 … 22085693	0	0	0	6	23
*Rcr9*/A08	Bra020936	10295068 … 10295637	0	0	0	58	93
	Bra020861	10809433 … 10825238	0	0	0	299	617

^1^SeqMan Pro software was used to sort variants that affect amino acid sequences into four groups: non-synonymous (NS), nonsense, frameshift and synonymous (Sy) variants. However, nonsense and frameshift variants were not found in all of the TNL genes in this study.

*Rcr4* was mapped into chromosome A03 in the genetic region of 267.48 to 310.75 cM (Table 3), corresponding to the physical region at A03_22692045 to A03_25649385 base, spanning about 2.96 Mb and including 441 genes based on the *B*. *rapa* reference genome. Six genes (*Bra012541*, *Bra019413*, *Bra019412*, *Bra019410*, *Bra019409* and *Bra019273*) as shown in Table 4, encode TNL-class disease resistance proteins in this region. No poly variants were identified in *Bra019412* and *Bra019273*. A total of 13 poly variants were identified from the coding sequences in the rest of four TNL genes (Supplementary Table S4). Three non-synonymous poly variants were identified in *Bra019413* and one in *Bra019410*. Only synonymous SNPs were found in *Bra012541* and *Bra019409* (Table 4). These five genes locate on chromosome A03 from 23,717,282 from 25,352,019, in an interval of 1.63 Mb.

*Rcr8* was mapped in the region of 126.91 to 164.4 cM (Table 3), corresponding to a physical region at A02_18503233 to A02_22097179 base, spanning about 3.59 Mb and including 396 genes. Four genes (*Bra022069*, *Bra022071*, *Bra026556* and *Bra032996*) encode TNL-class disease resistance proteins in the interval (Table 4). However, no DNA variants were identified in the genes. These five genes are located on chromosome A02 from 18,690,084 from 22,085,693, in an interval of 3.40 Mb.

*Rcr9* was mapped in the region of 214.61 to 291.25 cM (Table 3), corresponding to a physical region at A08_7105657 to A08_13587639 base, spanning about 6.48 Mb and including 838 genes. Only two genes (*Bra020936* and *Bra020861*) are known to encode TNL-class disease resistance proteins in this region (Table 4). However, no poly variants were identified in the genes (Table 4). These two genes are located on chromosome A08 from 10,295,068 from 10,825,238, in an interval of 0.53 Mb.

## Discussion

GBS generates a wealth of short DNA sequence reads from random places in the genome. The current study characterized variants in the *B*. *rapa* population, identified SNP sites that can be used for genetic mapping, and detected QTLs for resistance to specific clubroot pathotypes. SNP variants were the most common types of DNA sequence variation, accounting for 88% of the variation in the *B*. *rapa* genome. The number of variants identified in each sample was correlated strongly with sequencing depth. These observations are consistent with a previous study using RNA-seq^33^. Although coverage was low in the current study, variants from the reference *B*. *rapa* genome^54^ were very frequent, with a mean of 55 K variants per plant in the BC_1_ population. One factor that likely contributed to this high frequency of variants was that the parental lines were canola, while the reference genome was a Chinese cabbage cultivar.

The construction of genetic maps in the early 1990s based on DNA markers led to an explosion of activity directed toward the identification of QTLs in crop species. In addition to phenotyping individual lines, QTL mapping usually requires the identification of genome-wide polymorphic DNA markers for linkage analysis. However, the process for screening and genotyping polymorphic markers through analysis of markers is labour-intensive and time-consuming^55^. Progress in next generation sequencing (NGS) allows the identification of DNA markers through GBS. In the current study, the susceptible parent, ACDC, was a doubled haploid line, so each SNP site from this line should have been homozygous. However, numerous heterozygous SNP sites were identified. After filtering out the false heterozygous SNP and monomorphic SNP sites, some of the SNP sites could not be assigned into the chromosomes of *B*. *rapa*. Similarly, only small portion of GBS SNP sites that could be used for QTL mapping was also observed from the previous study^44^. The causes of heterozygous SNP sites in ACDC and the SNP sites that could not be assigned into any chromosomes by JoinMap 4.1 are likely errors from DNA sequencing or alignment of short reads. NGS can generate large amounts of data with problems such as high per-base error rates and non-uniform coverage, together with platform-specific read error profiles and artifacts^56^. In addition, triplication occurs in the *B*. *rapa* genome^54^, which could result in an ambiguous alignment that potentially leads to biases and errors in variant assessment. Identifying and correcting these issues imposes several statistical and computational challenges for the reliable detection of variants from NGS data^56^.

The identification of SNPs for bi-parental mapping of QTL through GBS has been carried out in several crops^57–59^ including *B*.*oleracea* ^44^. After filtering, 1,584 polymorphic SNP sites distributed to 10 chromosomes of *B*. *rapa* were identified. In the current study, 92 BC_1_ plants were assessed with a mean of only 0.1-fold coverage. Despite this low coverage, a set of high quality SNP sites and three strong QTLs were identified, indicating that identification of SNP markers through GBS is a cost effective and efficient method for QTL mapping.

The clubroot-resistant canola cultivars in western Canada were released in 2009 and 2010. These cultivars exhibited strong resistance to pathotypes 2, 3, 5, 6 and 8 of *P*. *brassicae* present in Canada. However, resistance in all of the Canadian canola cultivars has been overcome by new strains of the pathogen identified in Alberta after three years cultivation. The breakdown of resistance could be due to resistance in the cultivars controlled by a dominant gene originating from the same source therefore new sources of resistance is urgently needed. Identification of three QTL in this study facilitates breeders to develop canola cultivars carrying multiple resistance genes with more durable resistance to clubroot.

Several recent studies in Canada have focused on identifying sources of clubroot resistance and molecular markers to transfer clubroot resistance into canola^18, 21, 33, 48–50^. Although at least six pathotypes of *P*. *brassicae* occur in Canada, the research has been focused on resistance to pathotype 3^21, 48–50^, which is the predominant pathotype on the Canadian prairies^9^. A clubroot resistance gene, *Rcr1*, with efficacy against pathotype 3, was mapped to chromosome A03 of *B*. *rapa* in the pak choy cultivar ‘Flower Nabana’^21^ and resistance to pathotypes 2, 5, 6 and 8 was associated with *Rcr1* region on chromosome A03^33^. A resistance gene on chromosome A08 of rutabaga (*B*. *napus* subsp. *napobrassica*) also conferred resistance to these five pathotypes^50^. In the current study, resistance to the five pathotypes was highly correlated and likely controlled by one gene or tightly linked cluster of genes when assessed using conventional genetic analysis. One co-localized major QTL, *Rcr4*, was identified using GBS. This supported the hypothesis that a single gene or tightly linked genes controlled resistance to the five pathotypes. Since single spore isolates of pathotype 5x were not available, a *P*. *brassicae* population L-G02 orginating from a clubbed root was used in this study. *P*. *brassicae* is assumed to exist as mixtures of pathotypes in the soil, and even in single clubbed roots different pathotypes have been detected^60^. However, different pathotypes were not identified based on Williams’ differential and the Canadian resistant cultivars^10^. Resistance to the novel pathotype 5x was not correlated with resistance to the other five pathotypes. Genetic analysis indicated that resistance to pathotype 5x was likely controlled by two unlinked genes (*Rcr8* and *Rcr9*) in the resistant line T19. These two loci for resistance to 5x were mapped onto chromosomes A02 and A08, respectively. This provides strong evidence that the two genes control resistance to pathotype 5x independently. Also, *Rcr9* on A08 appears to differ from the resistance gene on A08 identified in rutabaga, because *Rcr9* was effective against pathotype 5x but not against pathotypes 2, 3, 5, 6 or 8, while the resistance gene from rutabaga was effective against these five pathotypes^50^.

The effect of population size on QTL detection was conducted by Bradshaw *et al*.^61^. They evaluated floral morphology traits in monkey flower using populations of 93 and 465 F_2_ individuals. For QTLs common to the two populations, the estimate of effect size was reduced in the larger population. It is possible that the magnitude of QTL effects is overestimated in small populations. In this study, three strong QTL were identified by using the BC_1_ population consisting of 92 plants. Therefore, further studies on the effects of the *Rcr4*, *Rcr8* and *Rcr9* to clubroot resistance would be needed. A small F_2_ population with 78 plants was previously used for identifying QTL for resistance to clubroot in *B*. *oleracea* through GBS^44^.

Two cloned clubroot resistance genes, *CRa* and *Crr1*, have been reported previously to encode TNL proteins^52, 53^. Therefore, DNA changes in the TNL genes in the *Rcr4*, *Rcr8* and *Rcr9* target regions were examined. However, a precise determination of the candidate genes was not possible since very few variants were identified in the TNL genes. The reason for this is due to the low depth of sequencing in this study. In contrast, the candidate genes for *Rcr1* were well predicted based on DNA variants among the TNL genes in the target region through deep sequencing^33^. As the cost for NGS is declining, deeper sequencing will become more affordable so higher depth sequencing would be recommended in the future research. Nonetheless, we could further narrow the gene intervals from 2.96 to 1.63 Mb for *Rcr4*, 3.59 to 3.4 Mb for *Rcr8* and 6.48 to 0.53 Mb for *Rcr9* by identifying the TNL genes in the respective QTL regions. Clubroot resistance gene *Rcr1* was mapped to the same region as *Rcr4*, although *Rcr1* was identified in pak choy (*B*. *rapa* subsp. *chinensis*)^21, 33^ while *Rcr4* was identified from canola. Identifying the TNL gene(s) that correspond with *Rcr4* or *Rcr1*, and the relationship between *Rcr4* and *Rcr1* will be addressed after the genes have been cloned. Four TNL genes were found in the *Rcr8* region A02, spanning the interval of 18,503,233 to 22,097,179. In a previous study, *CRc* was mapped on A02 by QTL analysis and molecular marker m6R, which is located in the position of 2,112,653 to 2,113,153 of chromosome A02, and was shown to be closely linked to *CRc* ^30^. The difference in mapping location between *Rcr8* and *CRc* demonstrates that these two resistance genes are not allelic. Two TNL genes, *Bra020936* and *Bra020861*, were identified in the *Rcr9* interval. The cloned clubroot resistance gene *Crr1* is highly homologous to *Bra020861* in the *B*. *rapa* reference genome^53^. Identifying the TNL gene(s) that corresponds with *Rcr9* and the relationship between *Rcr9* and *Crr1* will also be addressed after the genes have been cloned.

## Materials and Methods

### Plant materials

T19, a *B*. *rapa* canola breeding line with clubroot resistance originating from the German turnip cultivar ‘Pluto’, was developed at the Saskatoon Research and Development Centre, Agriculture and Agri-Food Canada (SRDC, AAFC), Saskatoon, Saskatchewan, Canada. T19 was crossed to ACDC, a clubroot-susceptible doubled-haploid, self-compatible *B*. *rapa* canola breeding line also developed at the SRDC. The resulting F_1_ plants were backcrossed with ACDC to produce BC_1_. Plants in the BC_1_ population were self-pollinated to produce BC_1_S_1_. To overcome self-incompatibility in *B*. *rapa*, bud-pollination was performed or each stigma was sprayed with 3% salt solution to produce sufficient seeds for evaluation of plant reaction to pathotypes 2, 3, 5, 6, 8 and 5x of *P*. *brassicae*. Ninety two BC_1_S_1_ lines were assessed in the study.

### Isolates of *P*. *brassicae* and evaluation of BC_1_S_1_ population for resistance to clubroot

Similar method and experimental design as described by Suwabe *et al*.^31^ were used in this study. Twelve plants from each of BC_1_S_1_ lines, parental lines T19 and ACDC were tested for resistance to five single-spore isolates of *P*. *brassicae*, SACAN-ss3 (pathotype 2), SACAN-ss1 (pathotype 3), ORCA-ss4 (pathotype 5), AbotJE-ss1 (pathotype 6), and ORCA-ss2 (pathotype 8) under controlled conditions at the University of Alberta, Edmonton, Alberta, Canada. Resting spores of each isolate were extracted from the frozen galls as described^10^ and adjusted to a concentration of 5.0 × 10^7^ resting spores/mL for each of the isolates. One-week-old seedlings of the host genotypes, which were pre-germinated on moistened filter paper in Petri dishes, were inoculated by dipping the entire root system in the resting spore suspension for 10 sec. The inoculated seedlings were then immediately planted in 6 cm × 6 cm × 6 cm plastic pots filled with Sunshine LA4 potting mixture, with one seedling per pot. Pots were thoroughly watered and transferred to a greenhouse at 21 °C ± 2 °C with a 16-h photoperiod. The potting mixture in the pots was kept saturated with water for the first week after inoculation and then watered and fertilized as required. Also, the reaction of each BC_1_S_1_ line and the parental lines to the *P*. *brassicae* population L-G02^10^, representing pathotype 5x, was tested on 14 to 20 plants per line under controlled conditions at SRDC, following the method described^21^. The experiments for pathotype 5x were repeated twice with similar results and only the first experiment was used for correlation study and identification of QTL. A Canadian cultivar “45H29” that can differentiate pathotypes 5 and 5x was included in the pathology experiments related to pathotype 5x.

Six weeks after inoculation, the roots of each line were dug out, washed with tap water, and examined for club formation. Clubroot severity was evaluated on a 0 to 3 scale as described previously^62^, where 0 = no clubbing, 1 = a few small clubs, 2 = moderate clubbing, and 3 = severe clubbing. A disease severity index (DSI) was calculated for each host line, using the method of Horiuchi and Hori^63^ as modified by Strelkov *et al*.^6^:

$$\mathrm{DSI}\,( \% )=\frac{{\sum }^{}({\rm{rating}}\,{\rm{class}})\times (\#\,{\rm{plants}}\,{\rm{in}}\,{\rm{rating}}\,{\rm{class}})}{{\rm{total}}\,\#\,{\rm{plants}}\,{\rm{in}}\,{\rm{treatment}}\times 3}\times 100$$

Correlation coefficients among the DSIs values in BC_1_S_1_ families to six pathotypes of *P*. *brassicae* were calculated using Microsoft Excel. The significance of the correlation coefficients was determined though t-tests^64^.

Based on our previous observation^33^, BC_1_S_1_ lines with DSI < 60% were likely from resistant BC_1_ plants. We therefore classified BC_1_S_1_ lines with DSI < 60% as R and those lines with DSI > 60% as S lines in this study.

### DNA sequencing and alignment of reads to reference genome

DNA was extracted from young leaves of each of the 92 BC_1_ plants and parental lines following DNeasy Plant Mini Handbook (QIAGEN). The DNA samples from the 92 BC_1_ plants and 2 replications of the parental lines T19 and ACDC were sequenced by an Illumina HiSeq 2000 SE single-end lane at Data2Bio (Ames, IW, USA). The program SeqMan NGen 13 (DNASTAR, Madison, WI, USA) was used for short read assembly. Standard assembling and filtering parameters were used. Short reads from each of 92 BC_1_ samples and the combined two replicates of each parental line were aligned to the reference genome Brapa_sequence_v1.5.fa downloaded from: http://brassicadb.org/brad/downloadOverview.php. The reference genome consists of 10 chromosomes and 40,357 scaffolds. The total lengths of chromosomes and scaffolds are about 258 Mb and 27 Mb, equivalent to about 90% and 10% of the reference genome, respectively. To simplify data analysis, only the 258 Mb chromosome sequences were used in the current study.

### Identification of variants, variant filtering, construction of linkage map and QTL mapping

Discovery of variants (SNPs and InDels) in comparison with the DNA sequences in the *B*. *rapa* ‘Chiifu’^54^ from each BC_1_ sample was performed using SeqMan Pro 13 (DNASTAR, Madison, WI, USA). Comparison of the variants among the 92 BC_1_ samples was carried out using Qseq 13 (DNASTAR, Madison, WI, USA). Only SNPs were used for further examination. Detected GBS-SNP sites were named based on the reference chromosome and position on the reference chromosome sequences. A SNP site was called in a given sample at following criteria: depth >5, Q > 10 and SNP percentage >10%. The remaining SNP sites after filtering were further analyzed using JoinMap 4.1^65^. SNP alleles from the resistant parent T19 were scored as “h” and those from the susceptible parent ACDC as “a”. The Mendelian segregation distortion of each marker was examined using X^2^ test in JoinMap 4.1 and distorted markers were excluded from further analysis. Marker orders and positions in the genetic map were determined using maximum likelihood in Kosambi’s model with a minimum logarithm of odds (LOD) of ten. This set of SNP sites were used for interval mapping of QTLs for resistance to clubroot. A map was drawn using Mapchart 2.1^66^ based on the genetic location determined by JoinMap 4.1. Mapping of resistance to the six pathotypes was performed using MapQTL 6 (www.biometis.wur.nl) with the interval mapping method. The LOD score threshold was initially set at 3.0 for QTL declaration, and QTLs that exceeded this LOD threshold were considered as suggestive QTLs. If any relevant QTL was identified, the LOD score threshold was determined using the 1,000-permutation test with a confidence of 0.99. QTL with LOD scores greater than the thresholds 4.14, 4.09, 4.15, 4.61, 4.03 and 4.03 (Table 3) for resistance to pathotypes 2, 3, 5, 6, 8 and 5x, respectively, at a confidence of 0.99, were declared significant. The QTL effects were estimated as PVE by the QTL.

### Identification of DNA variants in the TNL genes in the target region

The poly variants that uniquely occurred in the R bulks but not in the S bulks with depth >5 in both samples were assessed further. Bulked segregant analysis was performed to identify DNA variants in the respective QTL intervals. Based on both phenotyping and genotyping results, selected samples of the 92 BC_1_ plants were classified as R or S. Short reads from R and S samples were pooled to form R and S bulks, and then each bulk read was aligned to the *B*. *rapa* reference genome. Identification of poly variants was carried out as described by Yu *et al*.^33^. Gene annotation was analyzed with Blast2GO^67^. The NBS-LRR genes described as Chalhoub *et al*.^68^ in the target region were also examined. Further confirmation of the genes with TNL domains was performed with *Arabidopsis thaliana* WU-BLAST2 Search at http://www.arabidopsis.org/wublast/index2.jsp.

## Electronic supplementary material

Supplementary tables and figures
