# Supplementary material for: Genotyping-by-sequencing reveals three QTL for clubroot resistance to six pathotypes of Plasmodiophora brassicae in Brassica rapa
Source: Sci Rep. 2017 Jul 3;7:4516. doi: 10.1038/s41598-017-04903-2 (PMC5495781; doi:10.1038/s41598-017-04903-2)
Supplement: Supplementary file 1 — Supplementary tables and figures [file 41598_2017_4903_MOESM1_ESM.docx]

**Genotyping-by-sequencing reveals three QTL for clubroot resistance to six pathotypes of *Plasmodiophora brassicae* in *Brassica rapa***

Fengqun Yu^1,^ *, Xingguo Zhang^1^, Gary Peng^1^, Kevin C. Falk^1^, Stephen E. Strelkov^2^, Bruce D. Gossen^1^

^1^Agriculture and Agri-Food Canada, Saskatoon Research and Development Centre, 107 Science Place, Saskatoon, SK, S7N OX2, Canada

^2^Department of Agricultural, Food and Nutritional Science, University of Alberta, Edmonton, AB T6G 2P5, Canada

*Correspondence and requests for materials should be addressed to F.Y. (email: fengqun.yu@agr.gc.ca)

**Table S1. SNP filtering and identification of SNP sites linked to the chromosomes of *Brassica rapa***

| Categories | Number of SNP sites | % |
| --- | --- | --- |
| ACDC false heterozygous | 3263 | 19.6 |
| Monomorphic | 8392 | 50.5 |
| SNP sites not assigned into 10 chromosomes | 3379 | 20.3 |
| SNP sites assigned into 10 chromosomes | 1584 | 9.5 |
| Total | 16618 | 100 |

**Table S2. Statistics of the linkage map of *Brassica rapa* consisting of 1584 SNP sites**

| Linkage group | No. of markers | Total length (cM) | Mean length (cM) |
| --- | --- | --- | --- |
| A01 | 189 | 519.4 | 2.75 |
| A02 | 176 | 508.02 | 2.89 |
| A03 | 146 | 416.77 | 2.85 |
| A04 | 127 | 438.85 | 3.46 |
| A05 | 127 | 263.15 | 2.07 |
| A06 | 155 | 397.11 | 2.56 |
| A07 | 182 | 564.32 | 3.1 |
| A08 | 143 | 568.98 | 3.98 |
| A09 | 219 | 709.51 | 3.24 |
| A10 | 120 | 416.42 | 3.47 |
| Total | 1584 | 4802.52 |  |
| Minimum | 120 | 263.15 | 2.07 |
| Maximum | 219 | 709.51 | 3.98 |
| Mean | 157 | 479.3 | 3.05 |

**Table S3. Average disease severity index (DSI) and associated SNP marker in R and S bulks.** "+" indicates the presence of SNP allele associated with R and "-" indicates the presence of allele associated with S. The BC_1_ plants for *Rcr8* R bulk did not contain A08_10272562 (+) and the BC_1_ plants for *Rcr9* R bulk did not containA02_18552018 (+).

| QTL loci | DSI or SNP allele | R bulk | S bulk |
| --- | --- | --- | --- |
| *Rcr4* | Pathotype 2 DSI | 27.9 | 98.8 |
|  | Pathotype 3 DSI | 23.3 | 98.5 |
|  | Pathotype 5 DSI | 26.6 | 99.4 |
|  | Pathotype 6 DSI | 25.4 | 99.1 |
|  | Pathotype 8 DSI | 26.7 | 99.8 |
|  | No. of lines with A03_23710236 (+) | 45 | 0 |
|  | No. of lines with A03_23710236 (-) | 0 | 41 |
| *Rcr8* | Pathotype 5x DSI | 32.4 | 98.6 |
|  | No. of lines with A02_18552018 (+) | 18 | 0 |
|  | No. of lines with A02_18552018 (-) | 0 | 16 |
| *Rcr9* | Pathotype 5x DSI | 36.3 | 98.6 |
|  | No. of lines with A08_10272562 (+) | 21 | 0 |
|  | No. of lines with A08_10272562 (-) | 0 | 16 |

**Table S4. Polymorphic SNPs identified from R bulks in the TIR-NBS-LRR genes in the *Rcr4* target region on chromosome A03**

| Gene | Position | Reference base | SNP | Impact |
| --- | --- | --- | --- | --- |
| *Bra012541* | 23719898 | G | A | Synonymous |
| *Bra012541* | 23719904 | T | C | Synonymous |
| *Bra012541* | 23719975 | G | A | Synonymous |
| *Bra012541* | 23720564 | C | T | Synonymous |
| *Bra019413* | 24353167 | C | T | Non-synonymous |
| *Bra019413* | 24353172 | T | A | Synonymous |
| *Bra019413* | 24353183 | C | T | Non-synonymous |
| *Bra019413* | 24353191 | C | G | Non-synonymous |
| *Bra019410* | 24378337 | C | G | Synonymous |
| *Bra019410* | 24378341 | T | A | Synonymous |
| *Bra019410* | 24378475 | G | A | Synonymous |
| *Bra019410* | 24378746 | T | G | Non-synonymous |
| *Bra019409* | 24383817 | C | T | Synonymous |


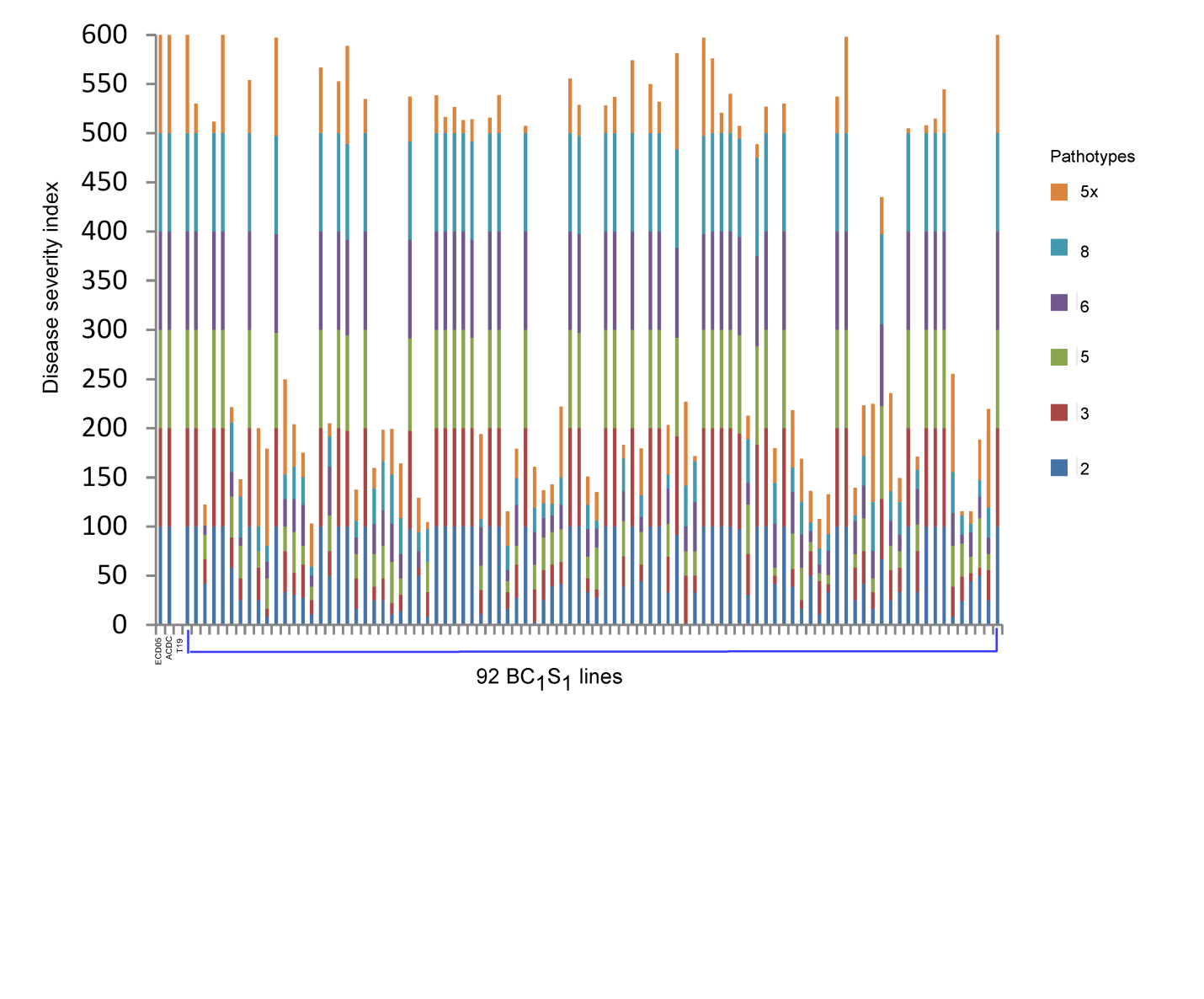


**Fig. S1. Disease severity indices of the parental lines, susceptible control ECD05 and the 92 individual BC_1_S_1_ lines with six pathotypes of *Plasmodiophora brassicae* found in Canada.**


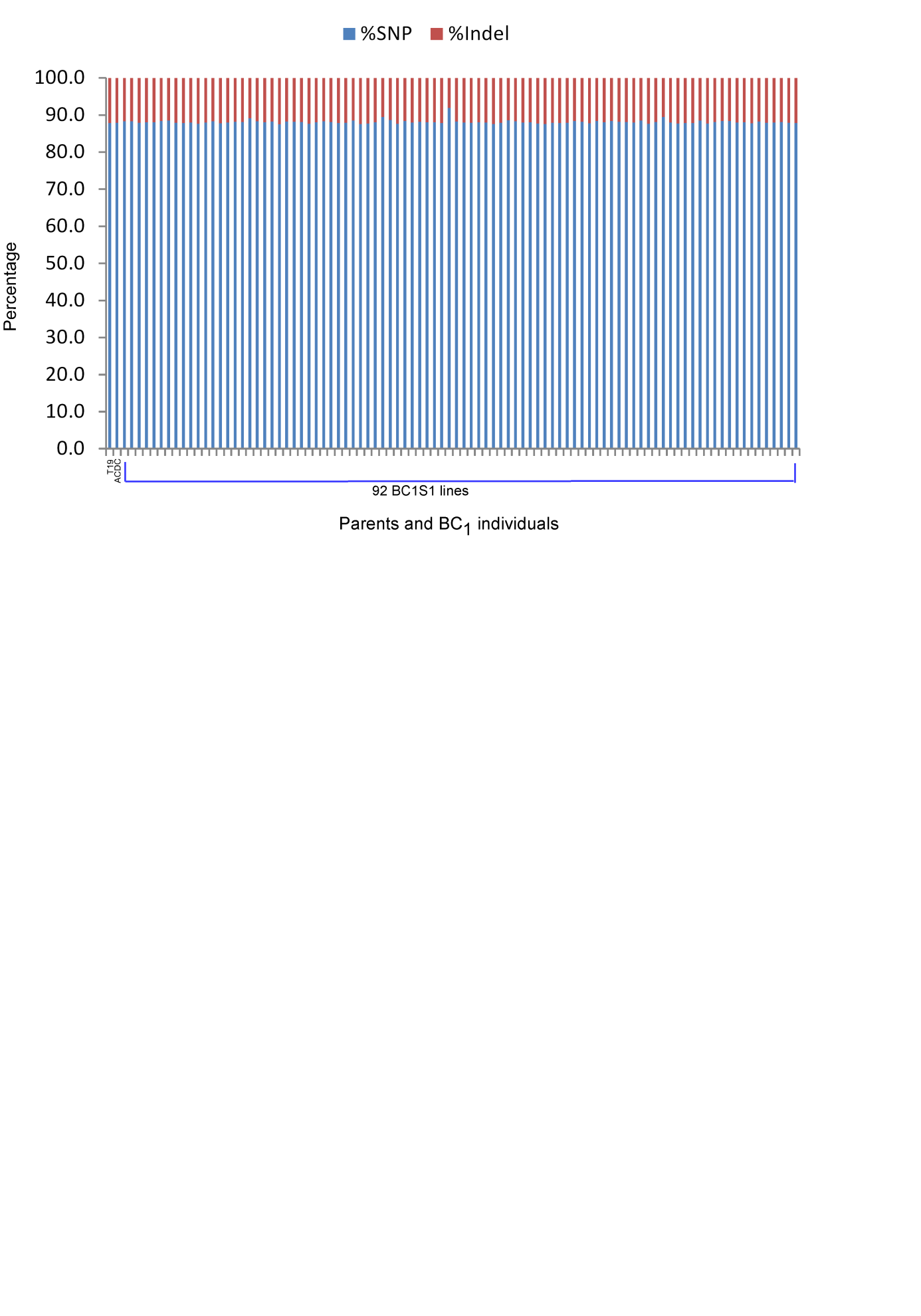


**Fig. S2. Proportion of SNPs and InDels in the parental linesT19 and ACDC, and 92 BC_1_ plants.**
